# Energy Landscape and Kinetic Analysis of Molecular Dynamics Simulations for Intrinsically Disordered Proteins

**DOI:** 10.1021/acs.jpcb.5c05390

**Published:** 2025-10-23

**Authors:** Moritz Schäffler, David J. Wales, Birgit Strodel

**Affiliations:** † Institute of Biological Information Processing, Structural Biochemistry (IBI-7), 28334 Forschungszentrum Jülich, 52428 Jülich, Germany; ‡ Yusuf Hamied Department of Chemistry, 2152 University of Cambridge, CB2 1EW Cambridge, U.K.; § Institute of Theoretical and Computational Chemistry, Heinrich Heine University Düsseldorf, 40225 Düsseldorf, Germany

## Abstract

Understanding the conformational dynamics of biomolecules requires methods that go beyond structural sampling and provide a quantitative description of thermodynamics and kinetics. For intrinsically disordered proteins (IDPs), energy landscape characterization is particularly crucial to unravel their complex conformational behavior. Here, we present a comprehensive protocol for analyzing molecular dynamics (MD) simulations in terms of energy landscapes, metastable states, and transition pathways. Our approach is based on the distribution of reciprocal interatomic distances (DRID) for dimensionality reduction, followed by clustering and kinetic modeling. Free energy surfaces and transition state barriers are computed directly from the simulation data and visualized using disconnectivity graphs. The method integrates two Python packages, DRIDmetric and freenet, with standard energy landscape tools based on kinetic transition networks, including PATHSAMPLE and disconnectionDPS. We demonstrate this workflow for simulations of the intrinsically disordered, aggregation-prone Alzheimer’s amyloid-β peptide in physiologically relevant environments. This modular framework offers a robust and interpretable way to extract thermodynamic and kinetic insights from MD data and is especially valuable for characterizing the diverse conformational states of IDPs.

## Graphical abstract

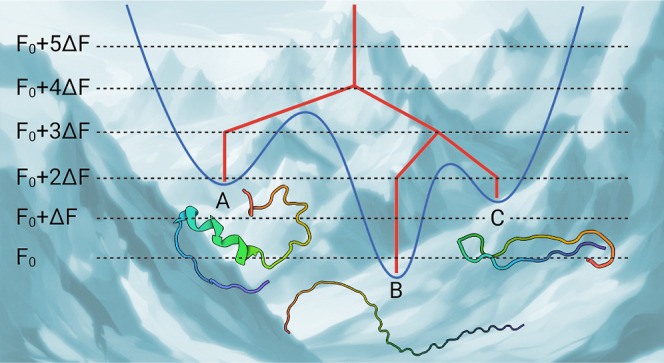

## 1 Introduction

Molecular dynamics (MD) simulations provide a powerful framework for exploring the structural and kinetic landscapes of protein conformational transitions. In particular, the concept of an energy landscape, mapping conformational states to their corresponding free energies, has proved essential for elucidating folding, misfolding, and self-assembly processes in both folded and intrinsically disordered proteins (IDPs).^1^ The amyloid-β (Aβ) peptide, a central player in the pathology of Alzheimer’s disease, represents a prototypical system for probing transitions between disordered and ordered states that drive toxic oligomer formation.^2,3^

In recent work by Schäffler et al.,^4^ the energy landscape of Aβ_42_, the Aβ variant with 42 amino acid residues, was characterized in detail using extensive MD simulations and structural clustering based on the distribution of reciprocal interatomic distances (DRID) metric. For the Aβ_42_ monomer, the landscape revealed a “structurally inverted funnel”, with disordered conformations occupying the global minimum. Upon dimerization, the landscape shifts to a more standard folding funnel culminating in β-hairpin formation. Using disconnectivity graphs^5,6^ and first-passage time (FPT) analysis,^7^ we identified distinct folding pathways and their associated time scales, highlighting the development of a salt bridge and cooperative binding through the formation of hydrophobic contacts as key mechanistic events in early oligomerization.

Building on these findings, this work presents a comprehensive guide to applying energy landscape theory and FPT-based kinetic analysis to MD simulation data. We outline the theoretical background, including: (i) DRID-based dimensionality reduction and clustering; (ii) free energy estimation from state populations and transition statistics; (iii) visualization of free energy surfaces via disconnectivity graphs; and (iv) calculation and interpretation of FPT distributions. A protocol-style implementation is provided to facilitate the application of this framework to diverse systems.

As a case study, we demonstrate the extended application of the method to Aβ_42_ in contact with physiologically relevant interaction partners. Specifically, we analyze simulations of the Aβ_42_ monomer in the presence of 1-palmitoyl-2-oleoyl-*sn*-glycero-3-phosphocholine (POPC) lipids and the glycosaminoglycan (GAG) chondroitin-4-sulfate with 8 subunits, two types of molecules known to modulate Aβ aggregation.^8^ For the interactions of Aβ_42_ with POPC, we consider only three lipid molecules forming a small cluster instead of larger assemblies like a lipid bilayer, inspired by the lipid-chaperone hypothesis that free lipids form complexes facilitating membrane insertion of Aβ and other amyloid proteins.^9^ This effect is based on a chemical equilibrium between dispersed lipids and their assemblies, characterized by the critical micellar concentration (CMC). Short-chain or charged lipids have CMCs in the μM range, while long-chain lipids have nM CMCs.^10,11^ Since these values resemble concentrations used in experiments and found in vivo, it is plausible that free lipid–protein complexes can form, influencing the structure of IDPs like Aβ_42_. By comparing energy landscapes and interconversion rates across these environments and with respect to neat solution,^4^ we assess how lipid and GAG interactions reshape folding funnels and transition barriers, providing insight into their roles in modulating the structural preferences of Aβ_42_. This work thus establishes a generalizable and robust methodology for energy landscape-based analysis of biomolecular folding and assembly, extending its applicability to simulation data across a broad range of molecular systems.

## 2 Theory and Methods

### 2.1 Molecular Dynamics Simulations

The Aβ_42_ peptide was modeled in all simulations with neutral histidine residues and without terminal capping groups, resulting in a net charge of −3. In this study, we consider two systems: Aβ_42_ in the presence of a glycosaminoglycan (GAG) chain (Aβ-GAG), and Aβ_42_ in the presence of three POPC lipids (Aβ-POPC). These simulations were originally performed in the context of separate studies,^12,13^ and are employed here to test the unified energy landscape framework. Despite minor differences in simulation protocols, all systems share the same force field, ion concentration, and temperature, so the results are directly comparable.

All simulations were carried out using GROMACS 2018.^14^ The peptide was described using the CHARMM36m force field,^15^ which has proved suitable for modeling both monomeric Aβ and its aggregation behavior.^16,17^ POPC lipids were modeled using the CHARMM36 force field,^18^ while GAGs were parametrized using the CHARMM-GUI Glycan Reader & Modeler module,^19,20^ consistent with previous studies of Aβ-GAG interactions.^21^

System preparation followed a standardized protocol: solutes were placed in a rectangular simulation box with a minimum distance of 1.2 nm from any periodic boundary. The systems were solvated with TIP3P water,^22^ and Na^+^ and Cl^–^ were added to achieve a physiological salt concentration of 150 mM while also ensuring charge neutrality. After equilibration, production simulations were conducted under *NpT* conditions at 1 bar using the Parrinello–Rahman barostat.^23^ The Aβ-GAG and Aβ-POPC systems were each simulated at 310 K using a Nosé–Hoover thermostat.^24,25^ Periodic boundary conditions were applied in all directions. Electrostatic interactions were calculated using the particle-mesh Ewald method,^26^ and van der Waals and real-space Coulomb interactions were truncated at 1.2 nm. For integration, the leapfrog algorithm was used with an integration time step of 2 fs.^27^ Total simulation times were extended compared to earlier studies, resulting in an accumulated simulation length of 6 μs per system. Configurations were saved every 20 ps for subsequent analysis. All simulations were performed on the JURECA-DC supercomputing cluster.^28^

### 2.2 Distribution of Reciprocal Interatomic Distances Metric

To reduce the dimensionality of the high-resolution MD trajectories while preserving essential structural and kinetic features, we applied the DRID metric, which transforms each conformation into a low-dimensional structural fingerprint by capturing local structural environments around selected reference atoms.

To apply the DRID metric, two sets of atoms are defined: a set of *m* centroids C representing structurally important positions (typically selected C_α_ atoms), and a set of *N* reference atoms A , excluding atoms covalently bonded to the centroids. For each centroid i∈C , the distribution of reciprocal interatomic distances to atoms in A is computed, and the first three moments of this distribution are used to characterize the centroid environment. Each structure is thus represented by a 3*m*-dimensional feature vector composed of the following moments

1 $$\mu_{i}=\frac{1}{N-1-nb_{i}}\sum_{j=1}^{N}\frac{1}{d_{ij}}$$

2 $$\nu_{i}={\left[\frac{1}{N-1-nb_{i}}\sum_{j=1}^{N}{\left(\frac{1}{d_{ij}}-\mu_{i}\right)}^{2}\right]}^{1/2}$$

3 $$\xi_{i}={\left[\frac{1}{N-1-nb_{i}}\sum_{j=1}^{N}{\left(\frac{1}{d_{ij}}-\mu_{i}\right)}^{3}\right]}^{1/3}$$

where *d*_*ij*_ is the distance between centroid G$c_{i}\in C$ and atom $a_{j}\in A$ , and *nb*_*i*_ denotes the number of atoms covalently bonded to centroid *i*. The structural dissimilarity between two conformations *j* and *k* is then quantified by the DRID space distance metric

4 $$s_{jk}=\frac{1}{3m}\sum_{i=1}^{m}{\left[{\left(\mu_{i}^{\left(j\right)}-\mu_{i}^{\left(k\right)}\right)}^{2}+{\left(\nu_{i}^{\left(j\right)}-\nu_{i}^{\left(k\right)}\right)}^{2}+{\left(\xi_{i}^{\left(j\right)}-\xi_{i}^{\left(k\right)}\right)}^{2}\right]}^{1/2}$$

Using this distance metric, structures are grouped into discrete states via regular space clustering in DRID space, as implemented in the PyEMMA Python package.^29^ The resulting clustering ensures that members within each state exhibit high structural similarity, while the overall network of states retains the slow dynamics and transition pathways of the underlying MD trajectory. This kinetic consistency has been demonstrated in previous applications of the DRID approach.^30,31^

### 2.3 Free Energy and Transition State Estimation

The free energy landscape (FEL) of a molecular system encodes both its thermodynamic stability and kinetic behavior. In the present work, the FEL is constructed from the discrete state representation obtained via clustering in DRID space, where each cluster defines a minimum in the landscape. Transitions between these minima correspond to conformational changes observed during the MD trajectory.

The free energy *F*_*i*_ of each minimum (i.e., DRID state *i*) is calculated from its equilibrium occupation probability *p*_*i*_ via the relation

5 $$F_{i}=-k_{B}T\;\ln p_{i}$$

where *k*_B_ is the Boltzmann constant and *T* the absolute temperature. The occupation probabilities are estimated from the relative frequencies of state visits in the MD trajectory. To estimate the free energy barriers between states, we first construct the rate matrix *R* whose off-diagonal elements *r*_*jk*_ correspond to the observed transition rates from state *j* to state *k* in the state trajectory. We translate these rates into transition state free energies using the Eyring–Polanyi formulation

6 $$F_{jk}=F_{k}-k_{B}T\;\ln k_{jk}+k_{B}T\;\ln\left(\frac{k_{B}T}{h}\right)$$

where *k*_*jk*_ is the rate constant for the *j* → *k* transition, and *h* is Planck’s constant. Ideally, the forward and backward transition state energies should satisfy *F*_*jk*_ = *F*_*kj*_; however, due to finite sampling in MD simulations, this symmetry may not hold exactly. To obtain a consistent estimate of the transition state free energy, we average the forward and backward transition state free energies

7 $${\mathrm{F̂}}_{jk}=\frac{F_{jk}+F_{kj}}{2}$$

where ${\mathrm{F̂}}_{jk}$ represents the effective free energy barrier separating states *j* and *k*.

### 2.4 Visualization via Disconnectivity Graphs

To visualize the hierarchical organization of the free energy landscape retaining all degrees of freedom, without employing collective variables, we use disconnectivity graphs. These graphs represent the global connectivity between free energy minima by grouping them into superbasins according to their mutual accessibility through transition states below specified energy thresholds. Each local minimum, corresponding to a DRID-defined state, is depicted as a vertical line terminating at its respective free energy, while branches in the graph indicate a superbasin of minima connected through low-energy transition pathways, as shown in Figure 1. Starting from the lowest energy state, minima are progressively grouped into superbasins at increasing regular energy thresholds, spaced at intervals Δ*F*. When two or more minima become connected via a pathway with the highest transition state below the threshold, they are merged into a common branch. In this way, the tree-like structure of the disconnectivity graph reflects the topological organization of the FEL, enabling direct identification of folding funnels, energy barriers, and metastable basins. In this study, we use a threshold spacing of Δ*F* = 0.5 *k*_B_ *T*, which balances structural resolution and basin grouping at physiological temperature (310 K).

**Figure 1:**
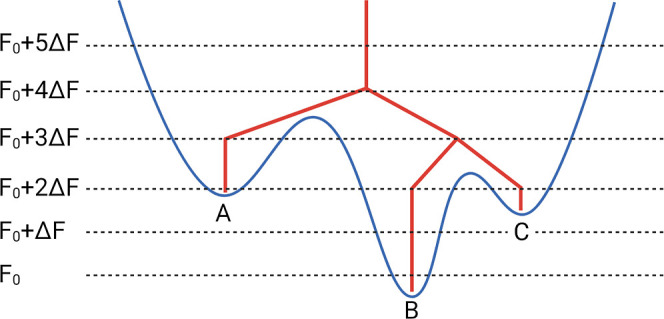
Schematic illustration of a disconnectivity graph. The graph (red) is constructed from a hypothetical free energy landscape (blue) comprising local minima A–C. Each minimum is represented by a vertical line terminating at the corresponding free energy.

Disconnectivity graphs provide a coordinate-independent representation of the FEL, including all degrees of freedom. For complex biomolecular systems meaningful reaction coordinates may be difficult to define, and we avoid projections along predefined coordinates. The minima are positioned on the horizontal axis so that low-lying states are placed in the middle of local funnel structures. This choice is designed to highlight the organization of the landscape.

### 2.5 First Passage Time Analysis

While the free energy landscape governs the structural and dynamical properties of a molecular system, experimental observables often correspond to relaxation times associated with specific conformational transitions. Studying the time scales of transitions between minima on the FEL can therefore bridge the gap between simulation and experiment and provide mechanistic insights into the underlying processes. These time scales can be quantified by the mean first passage time (MFPT), which is the average time required for the system to first reach a defined product state from a reactant state.

Beyond the MFPT, the full first passage time (FPT) distribution offers much more detailed information about the kinetic organization. Specifically, analysis of FPT distributions provides access to the topological structure of the energy landscape and enables the identification of distinct signatures associated with relaxation to different funnels or metastable states.^7^ We obtain the FPT distribution for the transition A ← B, from the reactant state B to the product state A, by treating A as an absorbing state, since the dynamics remain unchanged up to the point of absorption. Let I denote the set of intervening states, defined as I ≔ S\(B ∪ A), where S is the full state space. *P*_α_(*t*) represents the occupation probability of state α at time *t*. Then the time evolution of these probabilities for states in I ∪ B is governed by the master equation

8 $$\left[\begin{array}{l} {Ṗ}_{I}\left(t\right)\\ {Ṗ}_{B}\left(t\right) \end{array}\right]=\left[\begin{array}{ll} K_{II}-D_{I} & K_{IB}\\ K_{BI} & K_{BB}-D_{B} \end{array}\right]\left[\begin{array}{l} P_{I}\left(t\right)\\ P_{B}\left(t\right) \end{array}\right]=MP_{I\cup B}\left(t\right)$$

where **K**_*XY*_ is the rate matrix for transitions between sets of states *X* and *Y*, and **D**_*X*_ is a diagonal matrix containing the total escape rates for each state in *X*, i.e., ${\left[D_{X}\right]}_{ii}=\sum_{j}K_{ji}$ .

Solving this master equation via eigenvector decomposition yields an analytic expression for the first passage time distribution *p*(*t*)­

9 $$p\left(t\right)=\sum_{l}\nu_{l}e^{-\nu_{l}t}A_{l}$$

where ν_*l*_ > 0 are the eigenvalues of −**M**, and *A*_*l*_ are amplitudes determined by the corresponding eigenvectors. This expression describes the FPT as a weighted sum of exponential decay modes.

To identify competing relaxation time scales, the distribution is represented on a logarithmic time scale^7,32,33^ as P(y) with *y* = ln  *t*, yielding

10 $$P\left(y\right)=\sum_{l}\nu_{l}e^{y-\nu_{l}\;\exp\left(y\right)}A_{l}$$

This form reveals the presence of distinct relaxation modes in the system and allows identification of kinetic intermediates, fast and slow pathways, and dominant transition mechanisms. Peaks in P(y) correspond to characteristic time scales of relaxation processes between the defined states.

FPT analysis, when combined with energy landscape models, thus bridges thermodynamic and kinetic descriptions, enabling quantitative assessment of transition pathways and rates.

## 3 Practical Applications

The theoretical framework described above provides a powerful approach for extracting free energy surfaces, kinetic models, and transition pathways from full-dimensional MD simulations. In this section, we present a practical, protocol-style example demonstrating how to apply this methodology to simulation data.

Our implementation combines two Python packages, DRIDmetric and freenet, with the energy landscape tools PATHSAMPLE^34^ and disconnectionDPS^35^ based on analysis of kinetic transition networks. The DRIDmetric module performs dimensionality reduction on MD trajectories using the distribution of reciprocal interatomic distances (DRID), enabling the construction of state models that preserve both structural similarity and kinetic relevance. The freenet module then carries out regular space clustering in DRID space to identify metastable states, computes the corresponding transition matrix, and evaluates the associated free energies of the minima and transition states (i.e., energy barriers). The resulting kinetic model can be passed directly to PATHSAMPLE and disconnectionDPS to generate databases of transition pathways and construct disconnectivity graphs.

By integrating these tools, we provide a seamless analysis workflow—from raw MD trajectories to a comprehensive energy landscape representation, including thermodynamic basins, kinetic barriers, and relaxation time scales. This section outlines each step of the protocol, applying the methodology to representative simulation data of Aβ_42_ in the presence of either POPC lipids or a GAG molecule. These examples serve both as validation and as a guide for applying the framework to other biomolecular systems.

### 3.1 Installation

We recommend setting up a dedicated virtual environment (e.g., via venv or conda) to ensure compatibility and reproducibility of the analysis workflow. The required Python modules DRIDmetric and freenet can then be installed directly from their respective repositories using the following commands:
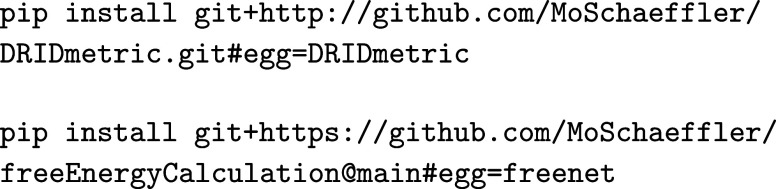


These packages provide the core functionality for dimensionality reduction, clustering, transition matrix construction, and free energy analysis. Both modules are compatible with Python 3.8 or higher and rely on standard scientific libraries, such as numpy, scipy, MDAnalysis, and deeptime. For more detailed installation instructions please refer to the appropriate github pages. The PATHSAMPLE and disconnectionDPS packages can be downloaded from https://www-wales.ch.cam.ac.uk/PATHSAMPLE/ and must be compiled locally as described at https://github.com/wales-group/examples.

### 3.2 DRIDmetric

The first step in constructing a kinetic model from MD simulation data is to reduce the dimensionality of the trajectory while preserving key structural and dynamical information. We use the DRIDmetric module to compute the distribution of reciprocal interatomic distances for each frame, resulting in a compact, low-dimensional representation of the system in DRID space. The DRID metric is computed based on a user-defined set of centroid atoms, which typically represent structurally relevant residues, and a reference set that defines the molecular environment. For the Aβ_42_ systems studied here, we select six C_α_ atoms corresponding to residues D1, F19, D23, K28, L34, and A42 as centroid atoms and the all peptide atoms as reference set, following our earlier work.^4^ Both selections use the syntax of MDAnalysis. Centroid selection is performed in a molecule-specific manner, either by identifying C_α_ atoms of residues directly relevant to the process under investigation or by uniformly spacing C_α_ atoms along the sequence. Although a more informed selection can improve sensitivity, subsequent analyses, such as free energy landscape reconstruction, have been demonstrated to be relatively robust with respect to the precise choice of centroids.^4^ Here is a minimal working example illustrating the computation of the DRID metric:
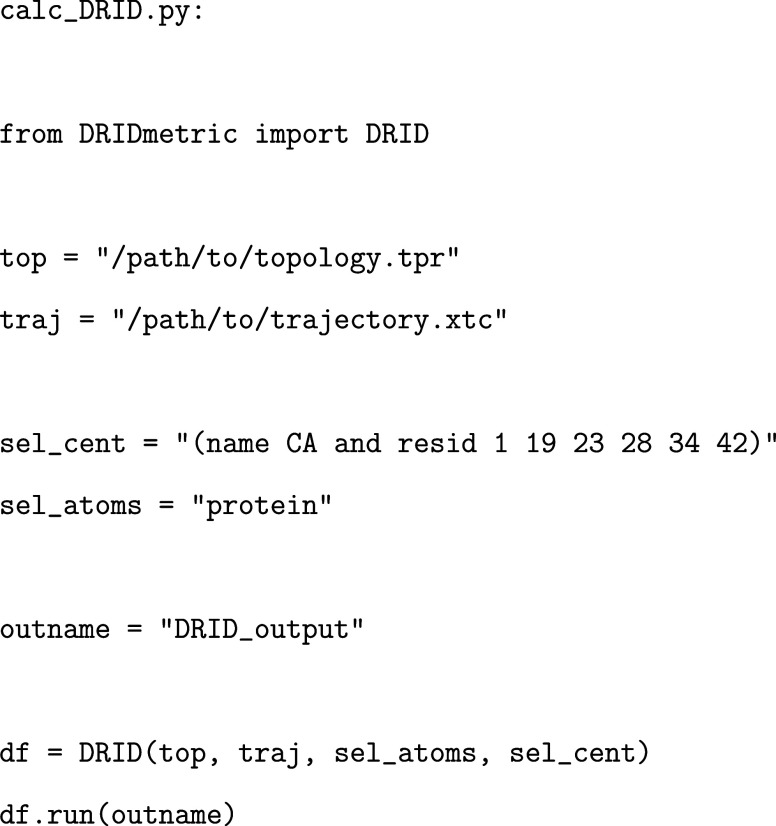


The output is a framewise DRID representation of the trajectory, stored as a .npy array, which serves as input for clustering and further kinetic analysis.

### 3.3 State Clustering and Free Energy Surface Construction

Following dimensionality reduction, the trajectory in DRID space is clustered into discrete conformational states, which form the basis of the kinetic model. We use the freenet module to perform regular space clustering, estimate the transition matrix, and compute the free energies of both states and transition barriers.

Given a precomputed DRID trajectory, clustering is performed with a user-defined cutoff (typically 0.02 nm^–1^ for Aβ_42_). The cutoff distance and the maximum number of centers (default: 2000) are key hyperparameters that define the granularity of clustering, with smaller values producing a finer partitioning of configurational space. For comparative analyses across multiple simulations, these parameters should be kept fixed to ensure that states of comparable entropy are identified consistently. Nevertheless, the cutoff yielding an optimal partitioning of the underlying FEL may be system dependent. Practical strategies to determine a suitable value include inspection of the ensemble of conformations grouped within a minimum or, more systematically, imposing a maximum RMSD criterion for structures within a single minimum. Equilibrium state probabilities and branching probabilities between directly connected states are then calculated, followed by the computation of free energies using the Eyring–Polanyi formulation. A minimal working example is shown below:
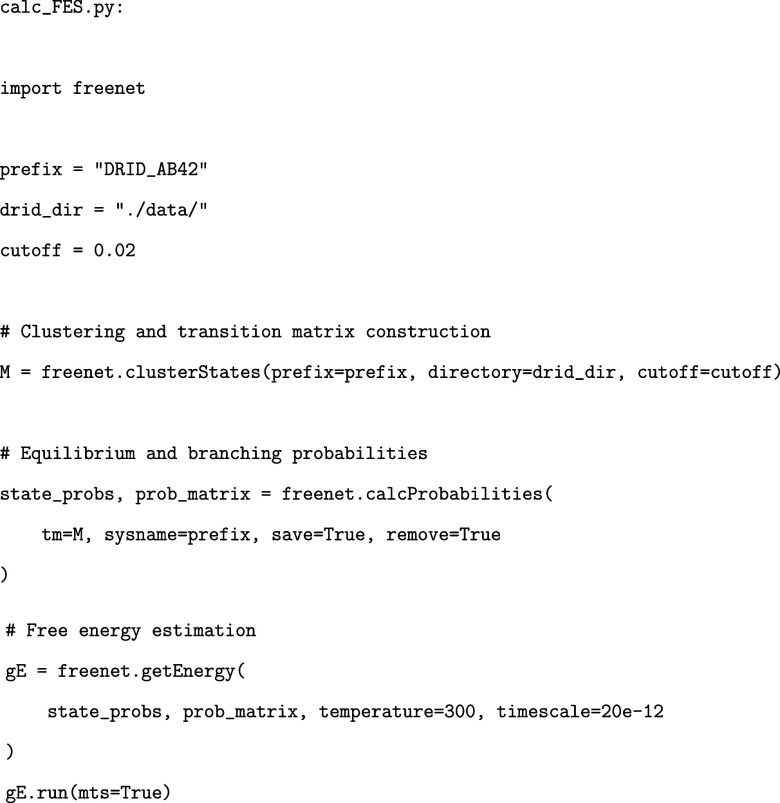


Here, the clusterStates function applies regular space clustering in DRID space, respecting the corresponding distance metric. In addition to generating the transition matrix, it produces a state assignment for each frame of the trajectory (i.e., the “state-trj”), linking each frame to its corresponding discrete state. It also outputs the deeptime clustering model as pickle file, which can be used to project additional trajectories into the same DRID-defined state space. This association enables direct backmapping of clustered states to structural configurations in real space, preserving interpretability of the free energy landscape in terms of molecular structure.

This workflow yields the free energy minimia and transition states whose configurations are saved in a format compatible with the PATHSAMPLE and disconnectionDPS tools, enabling subsequent pathway and landscape analysis.

### 3.4 Visualization of the Free Energy Landscape

Visualization of the database of free energy minima and transition state barriers as a disconnectivity graph is performed using the disconnectionDPS program. When executed in a directory containing the files min.data, ts.data, and a configuration file named dinfo, disconnectionDPS directly generates a graphical representation of the free energy surface as tree.ps. A minimal example of a valid dinfo configuration file is provided here:
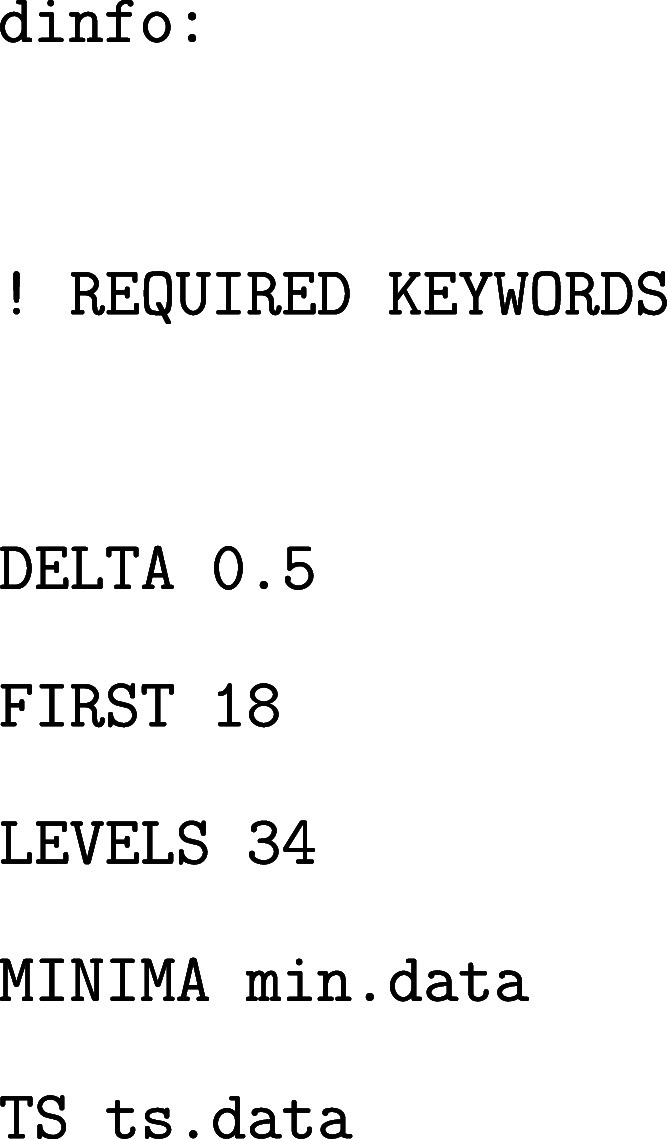


Here, DELTA specifies the energy threshold spacing (Δ*F*, in units of *k*_B_ *T*). The FIRST keyword sets the upper limit of the graph, which should be slightly higher than the highest transition state free energy to be included. LEVELS defines the number of energy levels to display in the disconnectivity graph. In general LEVELS should be slightly larger than FIRST/DELTA. The MINIMA and TS keywords specify the paths to the data files containing the minima and transition states, respectively. Additional keywords are available that automate some of these choices. Optional keywords can also be used to tailor the analysis and enhance interpretability of the FEL. For example, to identify and label the lowest-lying minima in the landscape, the following options may be added:
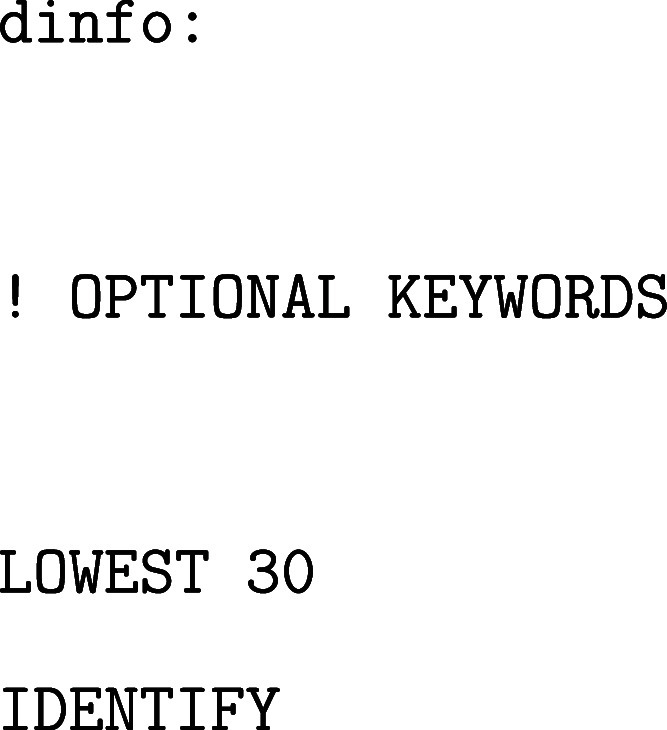


This configuration will produce a graph displaying only the 30 lowest minima, each annotated with its state ID, which is useful for identifying the global minimum and associated side funnels.

Moreover, if the state membership of each MD frame is known (e.g., from backmapping after DRID-based clustering), one can associate structural or dynamic observables with each state. These values can be visualized by color-coding the branches of the disconnectivity graph. In the example below, the average β-sheet content of each minimum is used as a coloring metric (order parameter):
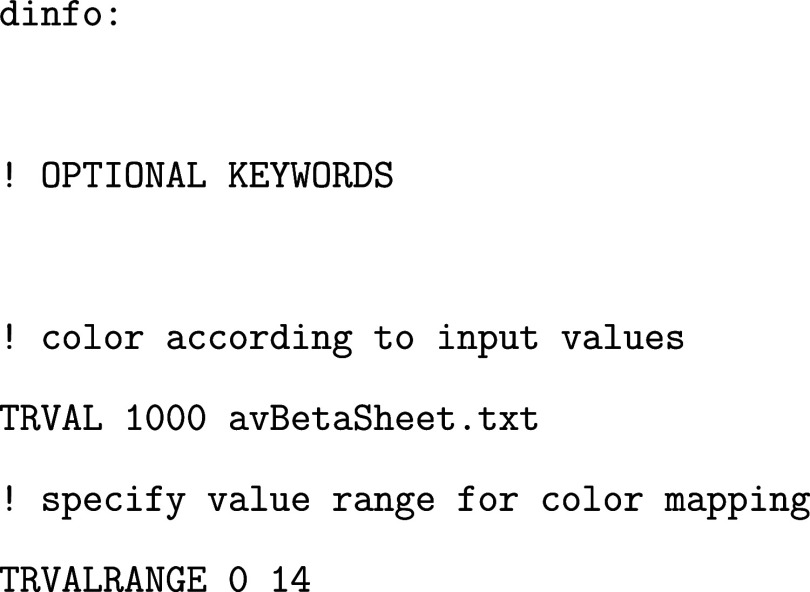


The file avBetaSheet.txt contains one scalar value per line, corresponding to the average observable (here the β-sheet content) for each minimum. Line *i* contains the value for minimum ID *i* (note that pathsample indexing begins at 1).

For more information on the full functionality of disconnectionDPS, please refer to the official documentation at https://www-wales.ch.cam.ac.uk/disconnectionDPS.doc/.

### 3.5 First Passage Time Analysis Using PATHSAMPLE

Assuming we have identified the global minimum of the free energy landscape (denoted *A*) and the minimum of a side funnel (*B*) based on an observable of interest, we can determine transition rates between these two states by calculating the first passage time (FPT) distribution using the PATHSAMPLE program.

In addition to the required min.data and ts.data files, PATHSAMPLE requires three files, a pathdata configuration file of directives, together with min.A and min.B files, which define the sets of minima associated with each end point. These files should contain the number of associated minima on the first line, followed by the minimum IDs on subsequent lines. For example:
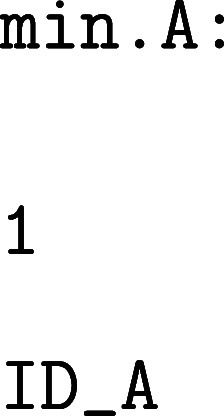


A minimal pathdata configuration file for computing the FPTs between minima *A* and *B* might look as follows:
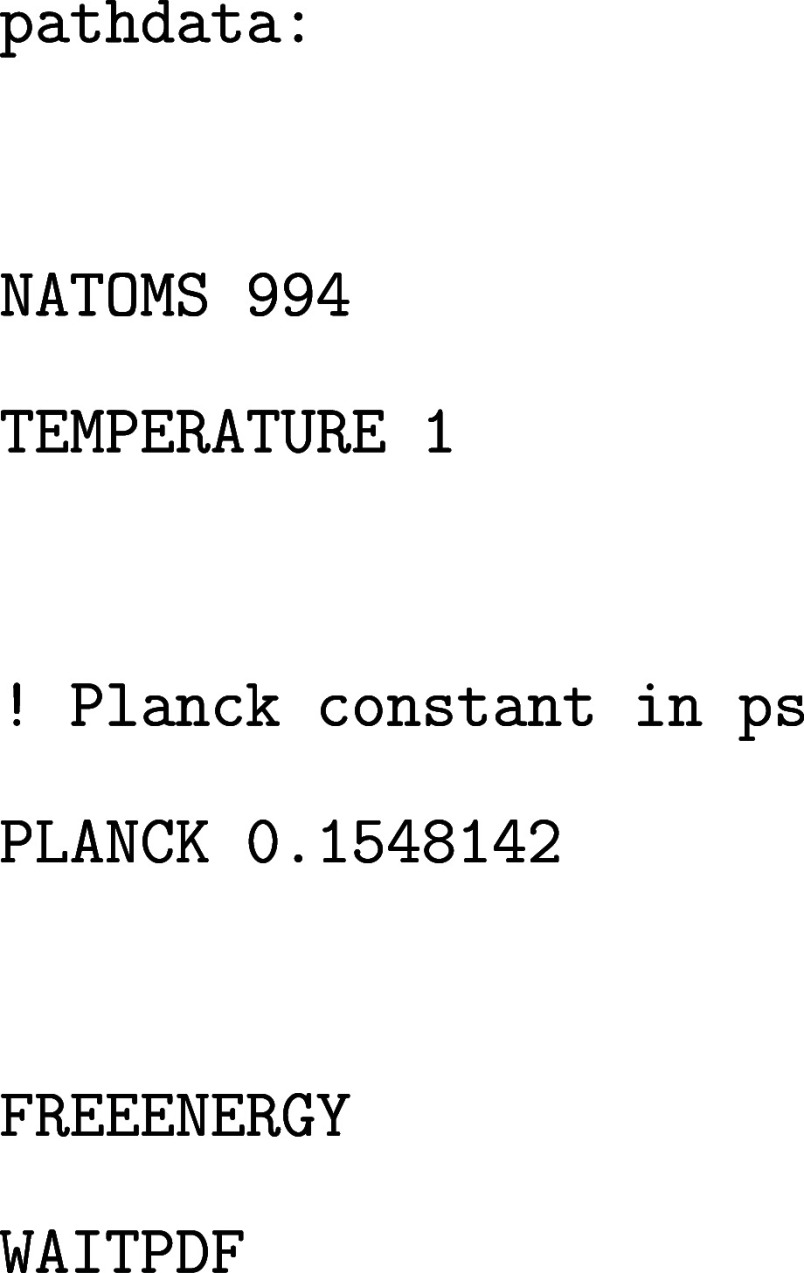


Running PATHSAMPLE from the terminal with this setup will compute the FPT distributions to the minima defined in min.A and min.B. The distribution for transitions from *A* to *B* is saved as waitlnpdfBA, while the reverse transition from *B* to *A* is saved as waitlnpdfAB.

Additionally, including the optional keyword WAITPDFPRINT in the pathdata configuration file will output all possible FPT distributions from every state in the network to the defined target minima. The corresponding files are named waitlnpdfB.ID and waitlnpdfA.ID, where ID refers to the starting minimum ID. The peak values of each FPT distribution, corresponding to the most probable transition times, are saved in the files peaksB.ID and peaksA.ID, respectively. Note that the FPTs are reported as natural logarithms of time, ln­(*t*); applying the exponential function yields the actual time scales in units of picoseconds.

## 4 Results

To demonstrate the utility of our methodology, we present comparative free energy landscape analyses of Aβ_42_ in neat solution, published before^4^ and serving as reference here, and in complex with the GAG molecule chondroitin-4-sulfate (with 8 subunits) and a POPC cluster composed of three lipid molecules. These evaluations highlight how our approach resolves subtle structural differences within aggregation-prone ensembles of Aβ_42_. Additionally, it enables mechanistic insights into environment-dependent folding pathways of IDPs, using Aβ_42_ as a case example. The FEL of the Aβ_42_ monomer in neat solution, previously analyzed in detail,^4^ is briefly revisited here to serve as a reference for comparison with the newly characterized systems.

### 4.1 Free Energy Landscape of the Aβ_42_ Monomer

Figure 2 shows the free energy landscape of the Aβ_42_ monomer, visualized as a disconnectivity graph. These data come from a previously published simulation^4^ and are revisited here to provide a point of comparison for the newly obtained results on Aβ_42_ in complex environments.

**Figure 2:**
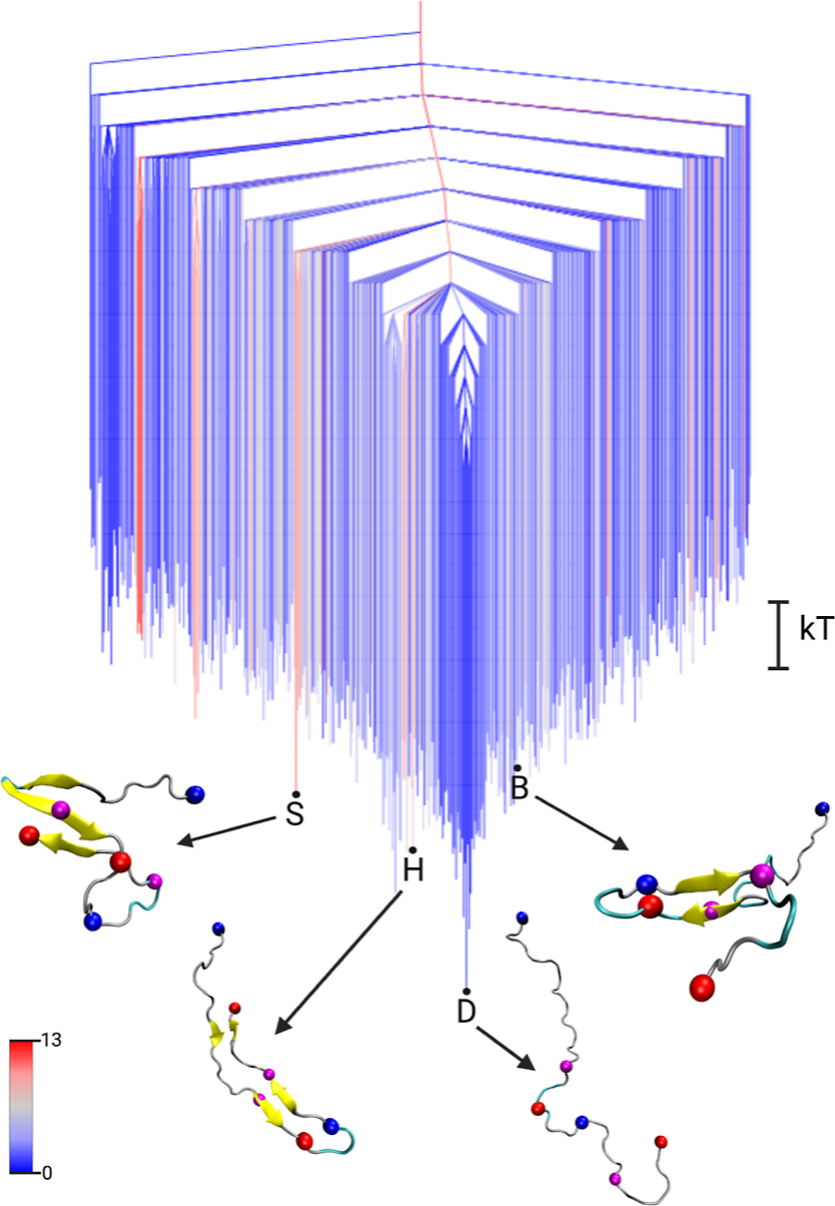
Free energy disconnectivity graph for the FEL of the Aβ_42_ monomer. The energies are given in units of *k*_B_ *T* (see scale bar on the right), with *k*_B_ the Boltzmann constant and *T* the absolute temperature. The branches are colored according to the average number of residues in β-sheet conformation in the ensemble of structures belonging to the corresponding minimum, ranging from blue (no β-sheets) to red (13 residues involved in β-sheets). Representative structures of some minima are shown, with D (for ‘disordered’) being the global minimum of the monomer FEL. The structures are shown in the cartoon representation, with β-sheets highlighted in yellow and the centroids used in the DRID metric shown as spheres (blue for positive charge at the N-terminus and K28 side chain, red for negative charge at the C-terminus and D23, magenta for the hydrophobic F19 and L34). Adapted from ref 4. Available under a CC-BY 3.0 Unported license. Copyright Moritz Schäffler, David J. Wales, and Birgit Strodel, 2024.

The monomer FEL exhibits a primary funnel leading to the global minimum. In contrast to typical folded proteins, this minimum corresponds to disordered conformations, denoted as state D (for “disordered”). Conformations featuring partial secondary structure, such as the β-hairpin states H and B characteristic for Aβ oligomers^36,37^ (state B is the global minimum of the dimer FEL identified in our previous work^4^) or the S-shaped motif typical of fibrillar forms,^38^ appear as excited states in the FEL, with free energy differences of Δ*F*_H_^mon^ = 2.3 *k*_B_ *T*, Δ*F*_B_^mon^ = 3.6 *k*_B_ *T*, and Δ*F*_S_^mon^ = 3.2 *k*_B_ *T*, respectively. Here, an ‘excited state’ refers to a higher energy free energy minimum. This organization of the energy landscape, in which disordered states reside at the bottom of the funnel, while structured conformations occupy higher-energy regions, has been previously referred to as an “inverted free energy landscape”.^39^ We suggested “structurally inverted funnel” or “disordered funnel” to emphasize that it is the structural ordering, rather than the topological shape of the funnel, that is inverted.

This baseline characterization of the monomer landscape serves as a reference for assessing how environmental factors, such as the presence of POPC lipids or GAGs, reshape the conformational preferences and folding kinetics of Aβ_42_.

### 4.2 Free Energy Landscape of Aβ_42_ in the Presence of a Glycosaminoglycan Chain

To illustrate the application of our framework to environmental perturbations of Aβ_42_, we computed the FEL of the peptide in the presence of a GAG chain. The analysis was performed using the same DRID metric as for the monomer, enabling direct comparison of the resulting energy landscapes. The same DRID metric as for the monomer in neat solution was employed to calculate the states, treating interactions between Aβ_42_ and the GAG only implicitly. Moreover, our previous study has revealed that due to minimal contacts between Aβ_42_ and the GAG molecule, there is no direct cooperative folding mechanism.^12^ Instead, the high negative charge of the GAG molecule alters the Na^+^ distribution within the system, leading to descreening of the intrapeptide electrostatic interactions and amplifying the hairpin-stabilizing D23–K28 salt bridge.

Figure 3 shows the FEL of Aβ_42_ in complex with a GAG chain, visualized as a disconnectivity graph. The landscape features a dominant funnel culminating in two competing β-hairpin structures. The global minimum, B_GAG_, consists of a compact hairpin configuration stabilized by an internal D23–K28 salt bridge and hydrophobic contacts involving the N-terminus. A slightly higher-lying minimum (Δ*F* ^GAG^ = 0.2 *k*_B_ *T*) corresponds to a more extended hairpin conformation, which is reminiscent of state B appearing as an excited state in the monomer FEL (Figure 2). However, the true state B can be found as a higher-lying excited state Aβ_42_-GAG FEL (Δ*F*_B_^GAG^ = 4.2 *k*_B_ *T*), while the disordered monomer minimum (state D) appears at Δ*F*_D_^GAG^ = 5.9 *k*_B_ *T*, embedded within a distinct side funnel of intrinsically disordered conformations. The presence of a defined side funnel leading to disordered states at higher free energies suggests a strong shift in the equilibrium ensemble of Aβ_42_ conformations toward more ordered states when in the neighborhood to a GAG molecule. The state with the highest β-sheet content, S_GAG_, adopts an S-shaped conformation (Δ*F*_S_^GAG^ = 1.3 *k*_B_ *T*), which has been previously associated with fibril formation.^38^ Its relatively low free energy supports the hypothesis that GAGs facilitate structural transitions relevant to aggregation.

**Figure 3:**
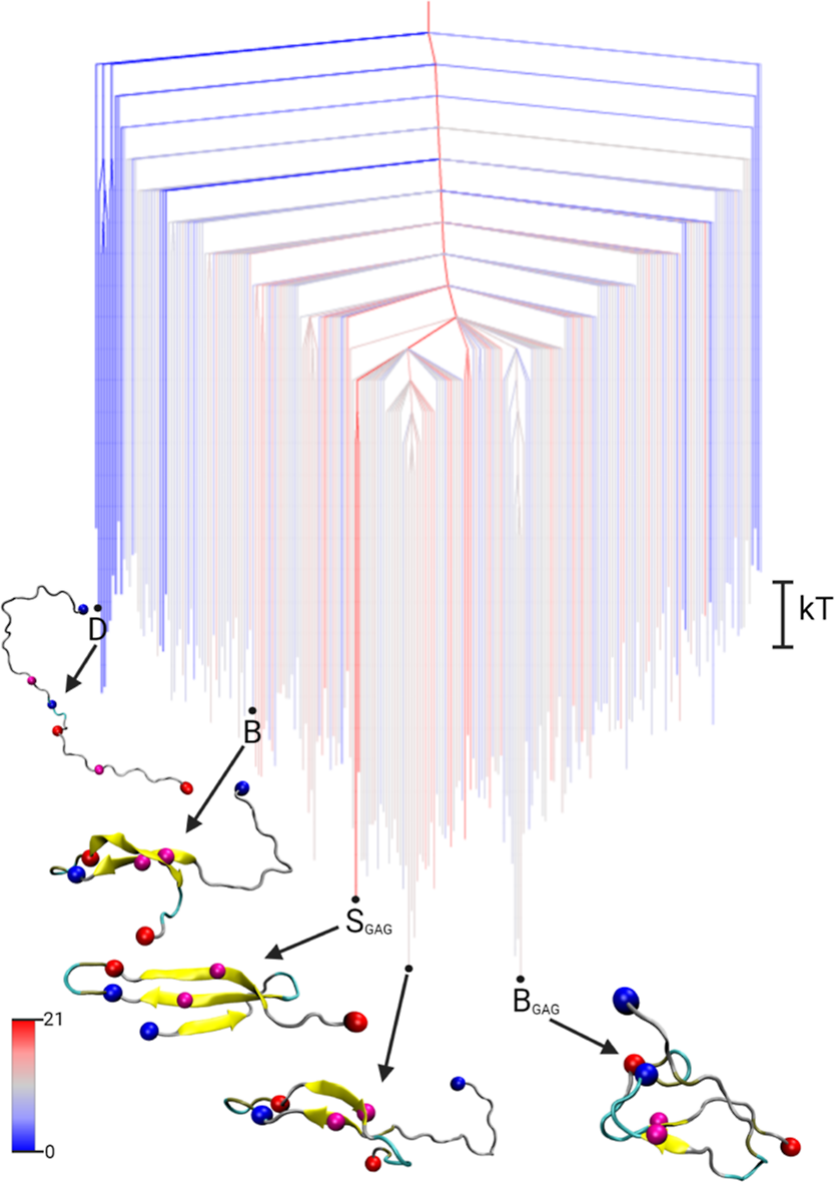
Free energy disconnectivity graph for Aβ_42_ in the presence of a GAG molecule. The energies are given in units of *k*_B_ *T* (see scale bar on the right), with *k* the Boltzmann constant, and *T* the absolute temperature. The branches are colored according to the average number of residues in β-sheet conformation in the ensemble of structures belonging to the respective minimum, ranging from blue (no β-sheets) to red (21 residues involved in β-sheets). Representative structures of some minima are shown, where B is the global minimum of the dimer FEL^4^ and D is the global minimum of the monomer FEL projected onto the Aβ_42_-GAG FEL. Furthermore, the global minimum B_GAG_ and the state with the highest β-sheet content S_GAG_ are highlighted. The structures are shown in the cartoon representation, with β-sheets highlighted in yellow and the centroids used in the DRID metric shown as spheres (blue for positive charge at the N-terminus and K28 side chain, red for negative charge at the C-terminus and D23, magenta for the hydrophobic F19 and L34). The GAG molecule is not shown as there are only transient contacts with the Aβ_42_ peptide.

Taken together, the FEL analysis reveals that in the presence of GAGs, Aβ_42_ preferentially adopts ordered β-hairpin conformations, with disordered states energetically disfavored. This supports experimental evidence that GAGs accelerate amyloid formation^40^ and identifies β-hairpin motifs as potential early stage aggregation intermediates promoted by the GAG environment.

### 4.3 Free Energy Landscape of Aβ_42_ in the Presence of POPC Lipids

To evaluate the influence of a lipid environment on the conformational ensemble of Aβ_42_, we applied our analysis framework to MD simulations of the peptide in complex with three POPC lipids. The same DRID metric used before was employed to define states, ensuring methodological consistency across the systems. This choice implies that the interactions with the lipid molecules are treated implicitly in the analysis via the peptide’s conformational response to the local lipid environment.

Previous work has shown that Aβ_42_ undergoes a disorder-to-order transition upon interacting with POPC lipids.^13^ At a 1:3 peptide-to-lipid ratio, simulations revealed competing structural tendencies: in some cases, a stable helix-kink-helix motif formed, while in others, β-sheet structures dominated. These transitions were found to depend on peptide–lipid contacts, particularly involving residues L17, A21, I32, and V36, which stabilize specific secondary structure motifs.

**Figure 4:**
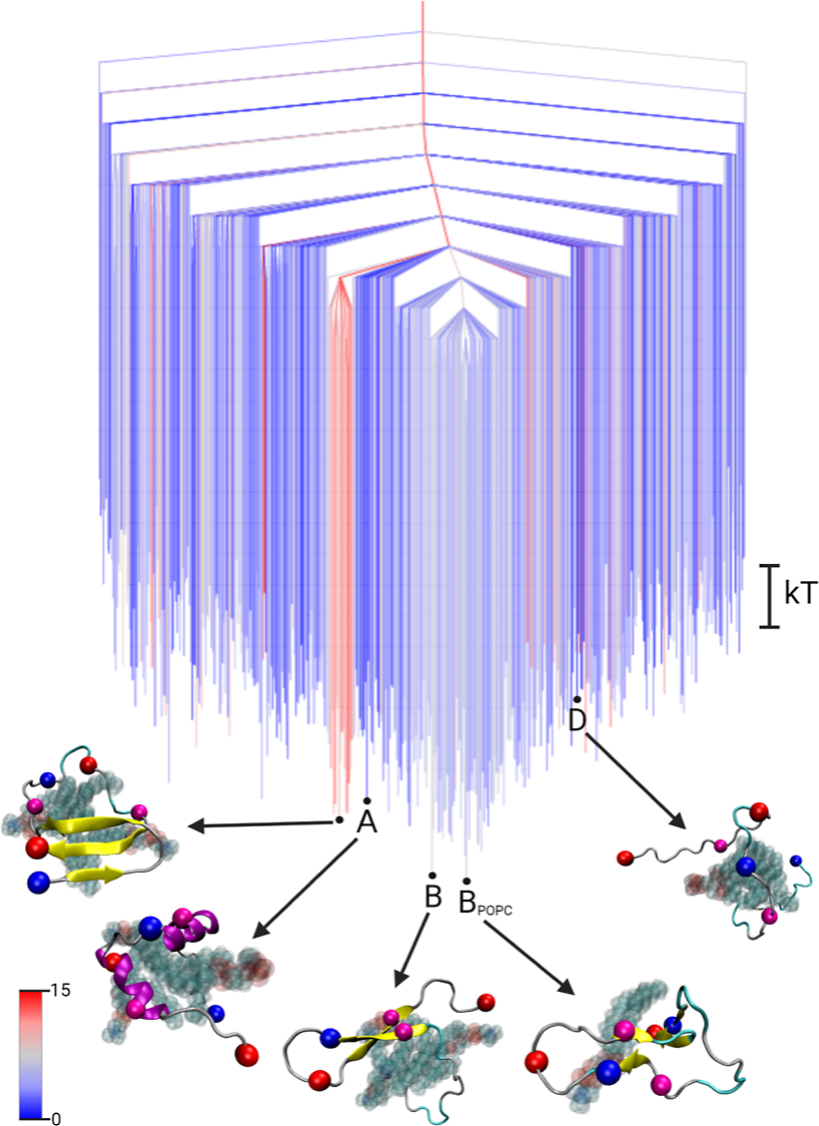
Free energy disconnectivity graph for Aβ_42_ in the presence of POPC lipids. The energies are given in units of *k*_B_ *T* (see scale bar on the right), with *k* the Boltzmann constant, and *T* the absolute temperature. The branches are colored according to the average number of residues in β-sheet conformation in the ensemble of structures belonging to the respective minimum, ranging from blue (no β-sheets) to red (15 residues involved in β-sheets). Representative structures of some minima are shown, where B is the global minimum of the dimer FEL and D is the global minimum of the monomer FEL projected onto the Aβ_42_-POPC FEL. The structures are shown in the cartoon representation, with β-sheets highlighted in yellow and the centroids used in the DRID metric shown as spheres (blue for positive charge at the N-terminus and K28 side chain, red for negative charge at the C-terminus and D23, magenta for the hydrophobic F19 and L34). The POPC lipids are shown as translucent spheres.

Figure 4 shows the FEL of the Aβ_42_-POPC system visualized as a disconnectivity graph. The landscape is characterized by a single broad funnel with a β-sheet-rich conformation at the global minimum (B_POPC_). This structure is distinct from the canonical β-hairpin observed in the dimer and GAG systems and suggests an alternate aggregation-prone topology driven by lipid interactions. The FEL is notably flatter than those observed for the other systems, as evidenced by the relatively small free energy differences between key states. Projection of the disordered monomer state (D) onto the POPC FEL reveals that it appears as a moderately high-energy local minimum (‘excited state’) with Δ*F*_D_^POPC^ = 3.0 *k*_B_ *T*, lower than its energetic offset in the dimer and GAG systems. This result reflects the flatter shape of the POPC landscape and suggests that disordered states remain accessible. Interestingly, the β-hairpin state B from the dimer FEL lies only Δ*F*_B_^POPC^ = 0.1 *k*_B_ *T* above the global minimum, indicating a high likelihood of formation. While previous studies observed a general β-sheet propensity of Aβ in the presence of lipid membranes,^41^ the specific hairpin topology had not been clearly resolved as a dominant structure. The low relative free energy identified here suggests, not only stability, but also kinetic accessibility in the presence of POPC.

Additionally, we identify a state with the highest α-helical content (S_A_), corresponding to the helix-kink-helix motif described in detail by Fatafta et al.^13^ and agreeing with structures found from NMR spectroscopy experiments of the micelle-bound Aβ peptide^42–46^ This helical structure appears at Δ*F*_A_^POPC^ = 1.0 *k*_B_ *T*, considerably lower than analogous α-helical conformations in the monomer and GAG systems, where helix formation is rare. The presence of such a low-energy helical state highlights the environment-specific modulation of the Aβ_42_ conformational ensemble by lipid interactions and reinforces the notion that POPC can stabilize distinct structural motifs.

In summary, the Aβ_42_-POPC FEL reveals a relatively flat landscape with multiple competing ordered states, including both β-sheet and α-helical motifs. The accessibility of these states underscores the conformational flexibility of Aβ_42_ in lipid-rich environments and supports the role of specific peptide–lipid contacts in guiding structural transitions relevant to early aggregation.

### 4.4 Time Scale Analysis from First Passage Time Distributions

To complement the structural characterization of the FEL, we analyzed the kinetics of state interconversion using FPT distributions calculated with PATHSAMPLE. Specifically, we focused on transitions between the disordered state (D) and the recurring β-hairpin state (B). For the Aβ_42_-GAG and Aβ_42_-POPC systems, we additionally analyzed the transitions to and from the respective global minima of their free energy landscapes. The resulting FPT distributions for the three systems under study are shown in Figure 5.

**Figure 5:**
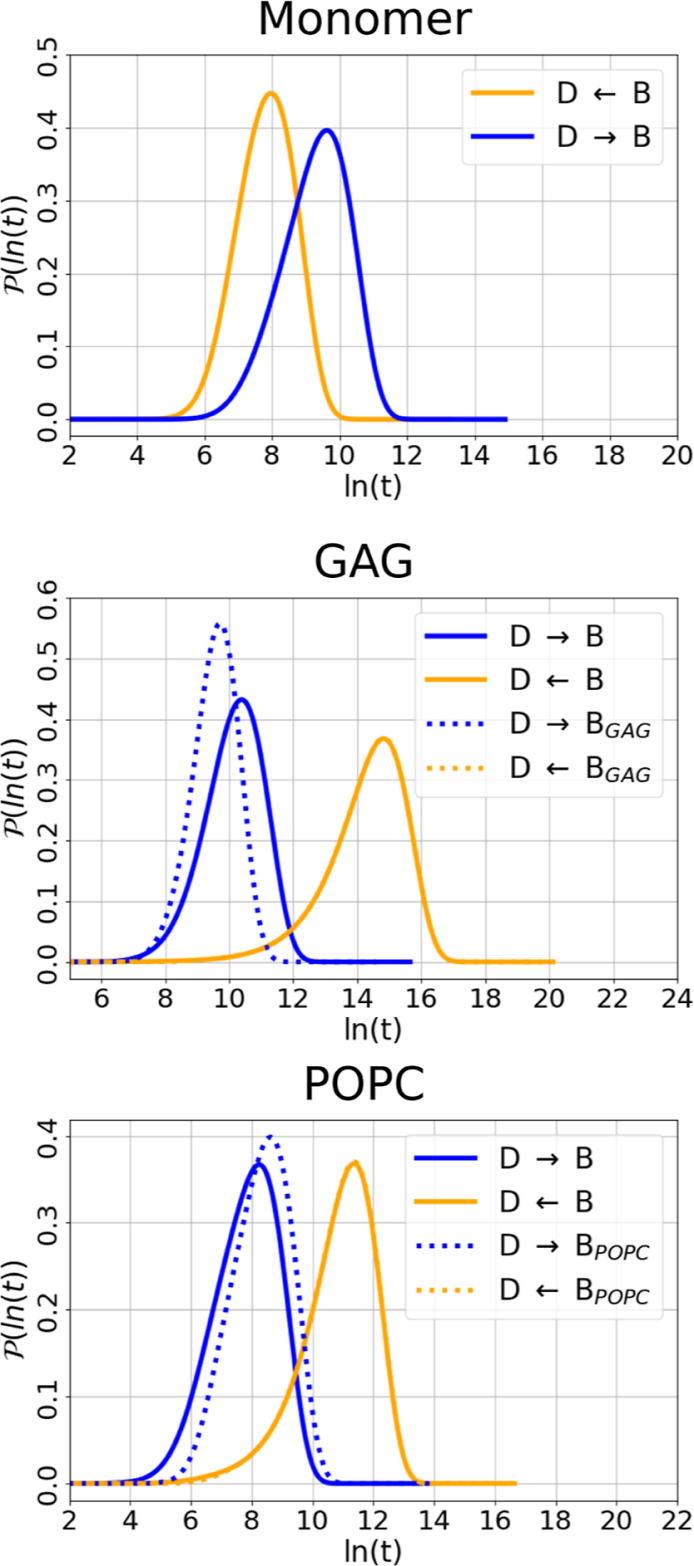
First passage time probability distributions for interconversions between disordered and β-hairpin states. The distribution P(ln⁡t) of first passage times *t* for transitions between the disordered state D and the β-hairpin state B is shown on a logarithmic scale. D and B were originally defined from the monomer and dimer FELs,^4^ respectively, and projected onto the Aβ_42_-GAG and Aβ_42_-POPC landscapes to identify structurally overlapping states. For the Aβ_42_-GAG and Aβ_42_-POPC systems, transitions to and from the global minima of the respective FEL are also included.

In the monomer, the FPT distributions feature well-defined peaks for transitions between D and B at time scales corresponding to $\tau_{D\to B}^{mon}∼15\;ns$ and $\tau_{B\to D}^{mon}∼3\;ns$ . Although the forward transition is five times slower than the reverse, both are relatively fast, indicating a shallow FEL with low kinetic barriers between states. In contrast, Aβ_42_-GAG exhibits a pronounced separation of time scales. Here, the transition times are $\tau_{D\to B}^{GAG}∼32\;ns$ and $\tau_{B\to D}^{GAG}∼2800\;ns$ . This clear imbalance indicates that once the system reaches state B, return to the disordered basin is highly unlikely on accessible MD time scales. Notably, the transition to the global minimum of the Aβ_42_-GAG FEL, a structurally distinct β-hairpin (B_GAG_), occurs even faster at $\tau_{D\to B_{GAG}}^{GAG}∼17\;ns$ . However, the return time to D from this state ( $\tau_{B_{GAG}\to D}^{GAG}∼2800\;ns$ ) remains long, confirming that disordered configurations are energetically disfavored in the GAG environment. Aβ_42_ interacting with POPC exhibits faster and more balanced transitions. The interconversion times between D and B are $\tau_{D\to B}^{POPC}∼4\;ns$ and $\tau_{B\to D}^{POPC}∼86\;ns$ . These values suggest that while the folding transition is comparatively fast compared to the other systems, the reverse transition is significantly faster than in the GAG systems, pointing to a shallower funnel and greater conformational flexibility, which is surprising as the Aβ_42_ forms a tight complex with the POPC lipids for the majority of the simulation time. Transitions involving the global minimum of the POPC FEL (B_POPC_) exhibit similar behavior, with $\tau_{D\to B_{POPC}}^{POPC}∼6\;ns$ and $\tau_{B_{POPC}\to D}^{POPC}∼86\;ns$ . The comparable time scales between D → B and D → B_POPC_ suggest that state B may act as an intermediate in the transition pathway toward the global minimum. The similar reverse times imply that both B and B_POPC_ reside within the same free energy basin.

The first passage time analysis complements the structural interpretation of the free energy landscapes by quantifying the kinetic accessibility of key states. The monomer and Aβ_42_-POPC systems display relatively flat landscapes with fast interconversion, while the Aβ_42_-GAG system, similar to the Aβ_42_ dimer,^4^ exhibits a steep, funnel-like kinetics favoring stable, folded states.

## 5 Discussion and Conclusion

In this study, we demonstrated the effectiveness and versatility of combining the DRIDmetric and freenet tools with the energy landscape exploration frameworks PATHSAMPLE and disconnectionDPS by applying this integrated workflow to simulations of the Alzheimer’s amyloid-β peptide. This application not only underscored the capability of our approach to extract meaningful thermodynamic and kinetic insights from MD data but also resulted in a practical, broadly applicable protocol. The protocol facilitates future studies of MD trajectories of IDPs and other aggregation-prone systems, promoting a standardized methodology for comprehensively characterizing their complex conformational energy landscapes. This approach should be applicable to biophysical processes that are accessible on a molecular dynamics time scale.

The case of Aβ_42_ illustrated that our energy landscape and kinetics analysis approach can uncover unprecedented details of its multifunneled energy landscape. As reviewed extensively, Aβ is polymorphic at multiple structural levels—monomer, oligomer, and fibril—owing to the inherent conformational ambiguity of the sequence.^3^ The ability to adopt a disordered state, especially in the N-terminal region resembling an IDP, as well as more ordered structures, such as α-helices and β-hairpins, reflects a highly dynamic conformational ensemble. These nearly equally probable structures, modulated by environmental factors, enable Aβ to transition between various states, facilitating its aggregation into diverse fibrillar forms.^47^ Our approach reveals the underlying complexity of the energy landsdcape, providing a framework to interpret Aβ’s polymorphism and its chameleon-like behavior, as it adaptively shifts conformation depending on the environment.^3^

In summary, the presented workflow, exemplified through the case of Aβ, highlights the potential to unveil detailed energy landscapes and kinetic pathways of IDPs and amyloid-forming systems. We suggest the broader application of this methodology to other IDPs and amyloid-related proteins, aiming to understand the conformational energy landscape at an atomic level of detail. Ultimately, this framework paves the way for more comprehensive insights into the structural basis of protein disorder and aggregation, with implications for therapeutic intervention and biomolecular design.
